# WT1 facilitates the self-renewal of leukemia-initiating cells through the upregulation of BCL2L2: WT1-BCL2L2 axis as a new acute myeloid leukemia therapy target

**DOI:** 10.1186/s12967-020-02384-y

**Published:** 2020-06-24

**Authors:** Bin Zhou, Xianghong Jin, Weiwei Jin, Xingzhou Huang, Yanfei Wu, Haiying Li, Weijian Zhu, Xiaoyi Qin, Haige Ye, Shenmeng Gao

**Affiliations:** 1 Laboratory of Internal Medicine, The First Affiliated Hospital of Wenzhou Medical University, 1 Xuefubei Street, Ouhai District, Wenzhou, 325000 Zhejiang China; 2 Department of Hematology, The First Affiliated Hospital of Wenzhou Medical University, 1 Xuefubei Street, Ouhai District, Wenzhou, 325000 Zhejiang China; 3 Department of Obstetrics and Gynecology, Wenzhou Hospital of Integrated Traditional Chinese and Western Medicine, Wenzhou, 325000 Zhejiang China

**Keywords:** Leukemia-initiating cell, Leukemia stem cell, Deubiquitinase inhibitor, Ubiquitin–proteasome signal, Wilms’ tumor-1, Self-renewal

## Abstract

**Background:**

Overexpression of Wilms’ tumor-1 (WT1) transcription factor facilitates proliferation in acute myeloid leukemia (AML). However, whether *WT1* is enriched in the leukemia-initiating cells (LICs) and leukemia stem cells (LSCs) and facilitates the self-renewal of LSCs remains poorly understood.

**Methods:**

MLL-AF9-induced murine leukemia model was used to evaluate the effect of knockdown of *wt1* on the self-renewal ability of LSC. RNA sequencing was performed on *WT1*-overexpressing cells to select *WT1* targets. Apoptosis and colony formation assays were used to assess the anti-leukemic potential of a deubiquitinase inhibitor WP1130. Furthermore, NOD/SCID-IL2Rγ (NSG) AML xenotransplantation and MLL-AF9-induced murine leukemia models were used to evaluate the anti-leukemogenic potential of WP1130 in vivo.

**Results:**

We found that *wt1* is highly expressed in LICs and LSCs and facilitates the maintenance of leukemia in a murine MLL-AF9-induced model of AML. WT1 enhanced the self-renewal of LSC by increasing the expression of *BCL2L2*, a member of *B cell lymphoma 2* (*BCL2*) family, by direct binding to its promoter region. Loss of *WT1* impaired self-renewal ability in LSC and delayed the progression of leukemia. WP1130 was found to modify the WT1-BCL2L2 axis, and WP1130-induced anti-leukemic activity was mediated by ubiquitin proteasome-mediated destruction of WT1 protein. WP1130 induced apoptosis and decreased colony formation abilities of leukemia cells and prolonged the overall survival in the THP1-based xenograft NSG mouse model. WP1130 also decreased the frequency of LSC and prolonged the overall survival in MLL-AF9-induced murine leukemia model. Mechanistically, WP1130 induced the degradation of WT1 by positively affecting the ubiquitination of WT1 protein.

**Conclusions:**

Our results indicate that *WT1* is required for the development of AML. WP1130 exhibits anti-leukemic activity by inhibiting the WT1-BCL2L2 axis, which may represent a new acute myeloid leukemia therapy target.

## Background

Acute myeloid leukemia (AML) is a fatal hematological malignancy characterized by the differentiation block and overproliferation of leukemic blasts. AML initiates from a small subset of leukemia stem cells (LSCs) that are responsible for chemotherapy resistance and relapse in AML patients [1]. LSCs have unlimited repopulating ability, prolonged residence in the G0/G1 phase of the cell cycle, and metabolism adaptations allowing evading the eradication by chemotherapy drugs [2]. With standard chemotherapy, only 40–50% AML patients (aged < 60 years) and about 15% AML patients (aged > 60 years) survive more than 5 years [3]. Therefore, developing effective targeted therapy for AML, especially targeting LSCs, is essential for improving the survival of AML patients.

The *Wilms’ tumor 1* (*WT1*) gene encoding a zinc finger transcription factor plays an important role in normal urogenital development and cancer pathogenesis [4, 5]. Although *WT1* is first identified as a tumor suppressor in Wilms’ tumor, emerging evidence indicates that *WT1* acts as an oncogene in various solid tumors and hematological malignancies [6]. The expression of *WT1* is increased in primary AML blasts compared with normal CD34^+^ hematopoietic stem and progenitor cells (HSPCs). Furthermore, higher expression of *WT1* in AML blasts correlates with worse clinical outcomes in AML patients [7]. As a transcription factor, *WT1* plays an important role in development, differentiation arrest, apoptosis, and proliferation [8].Overexpression of WT1 enhances cell proliferation and inhibits apoptosis through transcriptional activation of multiple oncogenes, such as *B*-*cell lymphoma*-*2* (*BCL2*) [9] and *cyclin D1* [10], and transcriptional repression of tumor suppressors, such as *E*-*cadherin* [11] and *cell division cycle 73* [12]. Additionally, overexpression of *WT1* sustains the survival of leukemia blasts [13]. For example, overexpression of *WT1* combined with *AML1*-*ETO*/*RUNX1*-*RUNX1T1* rapidly induces murine leukemia [14]. The knockdown of *WT1* expression by siRNA induces apoptosis and inhibits proliferation in leukemic cells [15]. More importantly, several compounds such as curcumin [16, 17] and HSP90 inhibitor 17-AAG [18] show strong anti-leukemic properties through the degradation of WT1 protein. Therefore, ectopic expression of *WT1* contributes to leukemogenesis and provides a potential candidate target for clinical intervention. However, the molecular mechanism by which WT1 facilitates the proliferation and self-renewal of LSCs remains to be elucidated.

WP1130 is a small molecular compound that was initially selected and identified for inhibiting Janus-activated kinase (JAK)-signal transducer and activator of transcription (STAT) pathway. The structure of WP1130 bases on AG490, the first compound used for inhibiting JAK2 kinase activity [19]. However, the subsequent experiments indicated that WP1130 acts as a cell-permeable deubiquitinating enzyme (DUB) inhibitor and fails to inhibit JAK2 kinase activity [20] directly. WP1130 inhibits proliferation and induces apoptosis in chronic myelogenous leukemia, hepatocellular carcinoma, or breast cancer [21–23]. WP1130 rapidly induces the accumulation of polyubiquitinated oncoproteins such as BCR-ABL [21], myeloid cell leukemia-1 (MCL1) [20], or MYC [24] into juxtanuclear aggresomes for degradation. It is, however, not clear whether WP1130 affects WT1 protein.

In the present study, we sought to investigate the function of *WT1* in LICs and LSCs. We found that *WT1* is required for the maintenance of AML by positively regulating *BCL2L2*. Furthermore, WP1130 exhibits strong anti-leukemic ability by suppressing proliferation, inducing apoptosis, and decreasing colony formation through inhibiting the WT1-BCL2L2 axis. Most importantly, WP1130 decreases the frequency of LSC and extends the survival time in *MLL*-*AF9*/*KMT2A*-*MLLT3*-induced murine leukemic model. Therefore, we conclude that the WT1-BCL2L2 axis plays an important role in the development of leukemia, and WP1130 has anti-leukemic potency by affecting WT1-BCL2L2 axis.

## Materials and methods

### Cell lines, primary AML blasts, and reagents

Human leukemia cell lines (THP1, K562, HL-60, and Kasumi-1, ATCC, Manassas, VA, USA) were purchased and cultured in RPMI 1640 supplemented with 10% fetal bovine serum (FBS, Invitrogen, Carlsbad, CA, USA) in humidified 37 °C incubator with 5% CO_2_. Bone marrow mononuclear cells (blasts % > 70%) from four AML patients with high *WT1* expressions were isolated by Ficoll density gradient centrifugation (GE Healthcare, Uppsala, Sweden). Human CD34^+^ umbilical cord blood (UCB) was obtained from the Translational Research Core of the First Affiliated Hospital of Wenzhou Medical University under an approved Institutional Review Board protocol. UCB and primary AML blasts were cultured in StemSpan Serum-Free Expansion Medium (SFEM, Stemcell Technologies, Vancouver, BC, Canada) supplemented with recombinant human stem cell factor (SCF, Stemcell Technologies), human fms related receptor tyrosine kinase 3 ligand (Flt3 ligand, Stemcell Technologies), human recombinant interleukin-3 (IL-3, Stemcell Technologies), human interleukin-6 (IL-6, Stemcell Technologies), and human thrombopoietin (TPO, Stemcell Technologies) at 10 ng/ml each. All procedures performed in studies involving human participants were following the ethical standards of the Ethics Committee of the First Affiliated Hospital of Wenzhou Medical University and the Declaration of Helsinki. All patients provided informed consent by the Declaration of Helsinki. The clinical characteristics of AML patients are summarized in Additional file 1: Table S1. WP1130 (MCE, Princeton, NJ, USA) and proteasome inhibitor MG132 (MCE) were dissolved in dimethyl sulfoxide (DMSO) and kept at − 20 °C until use.

### Blood smear and histology

Peripheral blood smears and bone marrow (BM) cytospins were stained by Wright-Giemsa stain using standard protocols [25].

### Engraftment of NOD/SCID-IL2Rγ mice (NSG)

Busulfan (30 mg/kg; B2635; Sigma) was intraperitoneally given to 8-week-old NSG mice (Shanghai Model Organisms Center, Shanghai, China) 1 day before xenotransplantation. THP1-GFP cells (2 × 10^6^) were intravenously injected into NSG mice. Two weeks of post-transplantation when the percentage of AML GFP^+^ cells in the blood reached 5%. Mice were divided into two groups (6 mice per group). One group was intraperitoneally injected with 50 μL DMSO/polyethylene glycol 300 (PEG300) (1:1 as the vehicle). Another group was intraperitoneally injected with WP1130 (40 mg/kg) in 50 μL DMSO/PEG300 [21]. Survival time was determined from the first day of the experiment until death. All the mice were housed in blanket cages with food and water available. All animal procedures and care are performed according to national and international policies and institutional guidelines of the First Affiliated Hospital of Wenzhou Medical University.

### MLL-AF9-induced murine leukemic model

BM cells were collected from tibia and femur from 8-week-old wild-type C57BL/6 J mice at 6 days after 5-fluorouracil (5-FU) treatment and resuspended in cold 1 × PBS buffer supplemented 2% FBS. BM cells were subjected to isolation of lineage-negative cells (Lin^−^) by harvesting the non-adherent fraction by selection with specific microbeads (CD11b^−^, Gr-1^−^, Ter119^−^, CD3^−^, B220^−^; Stemcell Technologies) following the manufacturer’s instructions. BM Lin^−^ cells were cultured in StemSpan SFEM (Stemcell Technologies) supplemented with murine SCF (10 ng/ml), TPO (50 ng/ml), and FLT3-L (50 ng/ml) overnight. The Lin^−^ cells were transduced with MSCV-GFP-IRES-MLL-AF9 (Addgene, Watertown, MA, USA) through two rounds of “spinoculation” as described previously [26, 27]. After transduction, Lin^−^ cells were intravenously injected into lethally irradiated C57BL/6 J mice (Shanghai Model Organisms Center, Shanghai). The mice were humanely sacrificed, and green fluorescent protein^+^ (GFP^+^) cells were sorted by flow cytometry when they developed full-onset leukemia.

### Bone marrow transplantation (BMT, in vivo reconstitution) assays

To determine the anti-leukemic effect of WP1130, we isolated BM GFP^+^ blasts (2 × 10^4^) from MLL-AF9-induced murine leukemia, and intravenously injected into lethally irradiated C57BL/6J mice, which were randomly divided into two groups: one group was intraperito-neally injected with 50 μL DMSO/PEG300 as vehicle group, and another group was intraperitoneally injected with WP1130 (40 mg/kg). In the secondary and tertiary BMT, sorted GFP^+^ cells from primary and secondary BMT receipts were collected and were injected into lethally irradiated secondary or tertiary recipient mice plus whole BM cells. The secondary or tertiary recipient mice were treated with or without WP1130. To determine the effects of *wt1* on MLL-AF9-induced murine leukemia, we sorted and transduced BM GFP^+^ cells from MLL-AF9-induced leukemia with sh-wt1 or sh-nc (negative control). After 4 days of puromycin treatment, transduced GFP^+^ cells were injected into lethally irradiated recipient mice. For secondary and tertiary BMT, BM GFP^+^ cells were isolated from primary and secondary BMT receipts. They were injected into lethally irradiated secondary or tertiary recipient mice plus whole BM cells. Overall survival was evaluated from the first day of the transplantation until death.

### Limiting dilution assays

BM GFP^+^ cells sorted by flow cytometry were isolated from secondary BMT recipients. Three different doses of donor cells were transplanted into lethally irradiated recipients for each group (n = 6). The numbers of recipient mice were counted only when they developed full-onset leukemia and died within 20 weeks post-transplantation. An extreme limiting dilution assay (ELDA) was used to assess the frequency of LSC [28].

### mRNA extraction and quantitative real-time PCR (qRT-PCR)

Total RNA was extracted by TRIzol (Invitrogen, Carlsbad, CA, USA) according to the manufacturer’s instruction. RNA concentration and quality were analyzed by measuring the absorbance at 260 nm with a spectrophotometer (DS-11, DeNovix, Wilmington, DE, USA). Total RNA was used as a template to synthesize cDNA for qRT-PCR analysis by ABI 7500 real-time PCR system (Applied Biosystems, Carlsbad, CA, USA). Relative expression was calculated using the $2^{-\Delta\Delta\text{CT}}$ method. The primer sequences were indicated in Additional file 4: Table S2.

### Other procedures

For details on Western blotting, apoptosis, CCK8, construction of plasmids, retrovirus production and cell transduction, co-immunoprecipitation (co-IP) assays, flow cytometry analysis, colony-forming assay, and RNA sequencing analysis please see Additional file 2: Materials and methods.

### Statistical analysis

Overall survival (OS) probabilities were estimated by the Kaplan–Meier method, and differences in survival distributions were compared using the log-rank test. OS was defined from the date of engraftment to death. For all the analyses, the *P* values were two-tailed, and a value *P *< 0.05 was considered statistically significant. All statistical analyses were performed using SPSS 22.0 (SPSS Inc, Chicago, IL, USA).

## Results

1. *wt1* is highly expressed in LICs and LSCs and facilitates the self-renewal of LSCs.
  To explore whether *wt1* is enriched in LICs or LSCs in comparison to normal HSPCs, the transcript of *wt1* was first measured in a murine MLL-AF9-induced AML model. Previous studies have shown that overexpression of *MLL*-*AF9* in murine HSPCs leads to AML [29]. We assessed the expression of *wt1* in immunophenotypic GFP^+^c-Kit^+^Mac-1^+^ cells as LICs [30], and L-GMP cells (GFP^+^Lin^−^c-Kit^+^Sca-1^−^CD34^+^CD16/32^+^) as LSCs [29]. *Wt1* expression was also assessed in Lin^−^ cells as normal comparable counterparts. The expression level of *wt1* was 4–sixfold higher in LICs and LSCs in comparison to normal Lin^−^ cells (Fig. 1a).

**Fig. 1:**
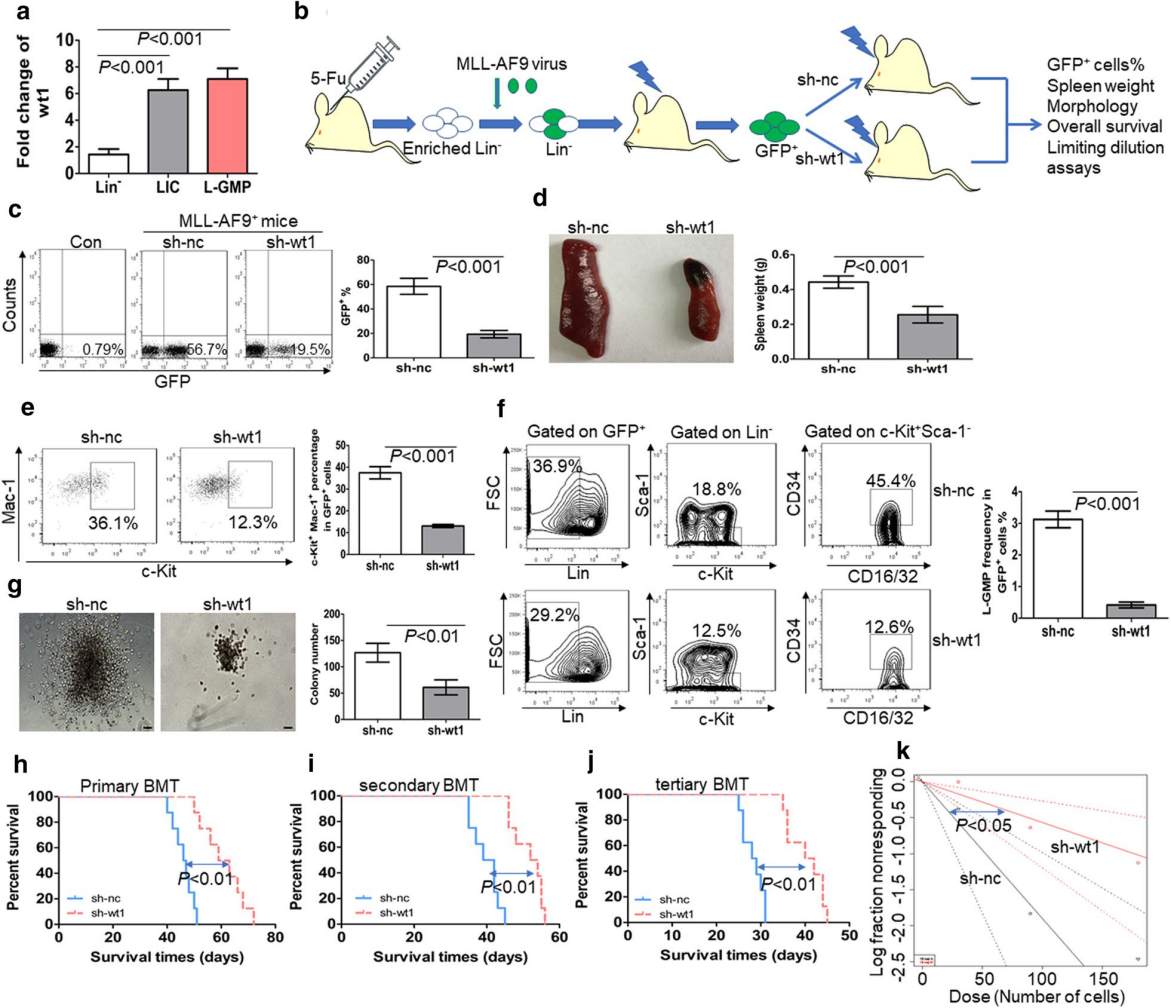
WT1 is required for the maintenance of MLL-AF9-induced murine leukemia. **a** The transcript expression of *wt1* is measured in LICs (GFP^+^c-Kit^+^Mac-1^+^) and LSCs (GFP^+^Lin^−^c-Kit^+^Sca-1^−^CD34^+^CD16/32^+^) from MLL-AF9-induced murine leukemia (n = 4) and BM Lin^−^ cells from normal C57/B6 mice (n = 4). **b** A schematic outline of the in vivo experiment using MLL-AF9-induced murine AML model. **c** The frequency of GFP^+^ cells was measured in BM cells from MLL-AF9-induced leukemia with knockdown of *wt1* (n = 4) or control *nc* (n = 4). Shown are the representative plots (left) and statistical analysis of the frequency of GFP^+^ cells (right). **d** A representative image of the spleen (left) and statistical analysis of spleen weight (right) in the MLL-AF9-induced murine leukemia with knockdown of *wt1* (n = 4) or control *nc* (n = 4). **e** Frequency of LICs was measured in GFP^+^ BM blasts from MLL-AF9-induced leukemia with knockdown of *wt1* (n = 4) or control *nc* (n = 4). Shown are the representative plots (left) and statistical analysis of the frequencies of c-Kit^+^Mac-1^+^ cells (right). **f** Percentage of L-GMP was assessed in BM GFP^+^ blasts from MLL-AF9-induced leukemia with knockdown of *wt1* (n = 4) or control *nc* (n = 4). Shown are the representative plots (left) and statistical analysis of the frequencies of L-GMP (right). **g** BM GFP^+^ cells were isolated from MLL-AF9-induced leukemia with knockdown of *wt1* (n = 4) or control *nc* (n = 4) and then plated on methylcellulose medium. Colonies were counted after 2 weeks (n = 4). Shown are the representative colony pictures (left) and statistical analysis of the number of colonies (right). Bar represents 50 µm. **h–j** Overall survival was determined in the primary BMT (**h**, n = 8), secondary BMT (**i**, n = 8), and tertiary BMT (**j**, n = 8) of MLL-AF9-induced murine leukemia with knockdown of *wt1* or control *nc*. **k** Limiting dilution assay of BM GFP^+^ cells from MLL-AF9-induced murine leukemia with knockdown of *wt1* (n = 6) or control *nc* (n = 6). The frequency of LSC and *P*-value were calculated by L-calc software
  To investigate whether the knockdown of *wt1* inhibits the self-renewal of LSCs, we isolated MLL-AF9-induced murine leukemic blasts, which were transduced with specific short hairpin RNA (shRNA) for *wt1* (sh-wt1) or control scrambled sh-nc. Transduced leukemic cells were transplanted into receipt mice (Fig. 1b). qRT-PCR indicated successful silencing of *wt1* in MLL-AF9-induced murine leukemia (Additional file 3: Fig. S1a). The frequency of GFP^+^ cells was substantially lower in BM cells with knockdown of *wt1* than those transduced with *nc* control (Fig. 1c). Furthermore, the percentage of AML blasts was lower in blood and BM cells with *wt1* knockdown in comparison to those transduced with *nc* control (Additional file 3: Fig. S1b). Besides, spleen weight was significantly decreased in leukemic mice transplanted with cells with knockdown of *wt1* in comparison to mice transplanted with cells transduced with scrambled *nc* control (Fig. 1d). To explore the relationship between *wt1* knockdown and the frequencies of LICs and LSCs, we measured the frequencies of c-Kit^+^Mac-1^+^ cells and Lin^−^c-Kit^+^Sca-1^−^CD34^+^CD16/32^+^ cells (L-GMP) in BM GFP^+^ cells from MLL-AF9-induced murine leukemia. The knockdown of *wt1* substantially reduced the frequencies of LIC (Fig. 1e) and LSC (Fig. 1f). GFP^+^ MLL-AF9 cells from BM were sorted for the evaluation of colony formation. The knockdown of *wt1* in MLL-AF9 cells significantly decreased the ability to form colonies by MLL-AF9 cells (Fig. 1g).
  To further clarify the function of WT1 in LSCs, we performed serial BMT with the same number of AML cells. The knockdown of *wt1* substantially extended the overall survival in primary, secondary, and tertiary BMT in comparison to control transduced MLL-AF9 cells (Fig. 1h–j). Besides, limiting dilution assays indicated that the estimated frequency of LSCs was significantly lower in murine leukemic blasts with knockdown of *wt1* in comparison to *nc* control (Fig. 1k and Additional file 8: Table S3, 1 in 177 versus 1 in 53, *P *< 0.05).
2. WT1 increases the expression of BCL2L2 by binding its promoter region.
  As a transcription factor, *WT1* regulates the expressions of target genes by binding to their promoter regions [31]. To determine the potential targets of *WT1* in leukemic cells, we transduced U937 cells in the absent expression of *WT1* [32] with retrovirus vector MSCV-WT1, which expresses *WT1* isoform (Ex5-/KTS-). RNA sequencing analysis was performed to select the potential genes regulated by *WT1*. A total of 118 genes were significantly increased (> twofold), and 339 genes (< − twofold) were decreased in U937 cells overexpressing WT1 in comparison to cells transduced with a vector encoding for *nc* control (Fig. 2a, b). Gene Set Enrichment Analysis (GSEA) demonstrated that negative regulation of myeloid leukocyte differentiation was enriched in U937 cells overexpressing WT1 (Fig. 2c). Furthermore, genes related to hematopoietic cell lineage were negatively enriched in WT1-overexpressing U937 cells (Fig. 2d).

**Fig. 2:**
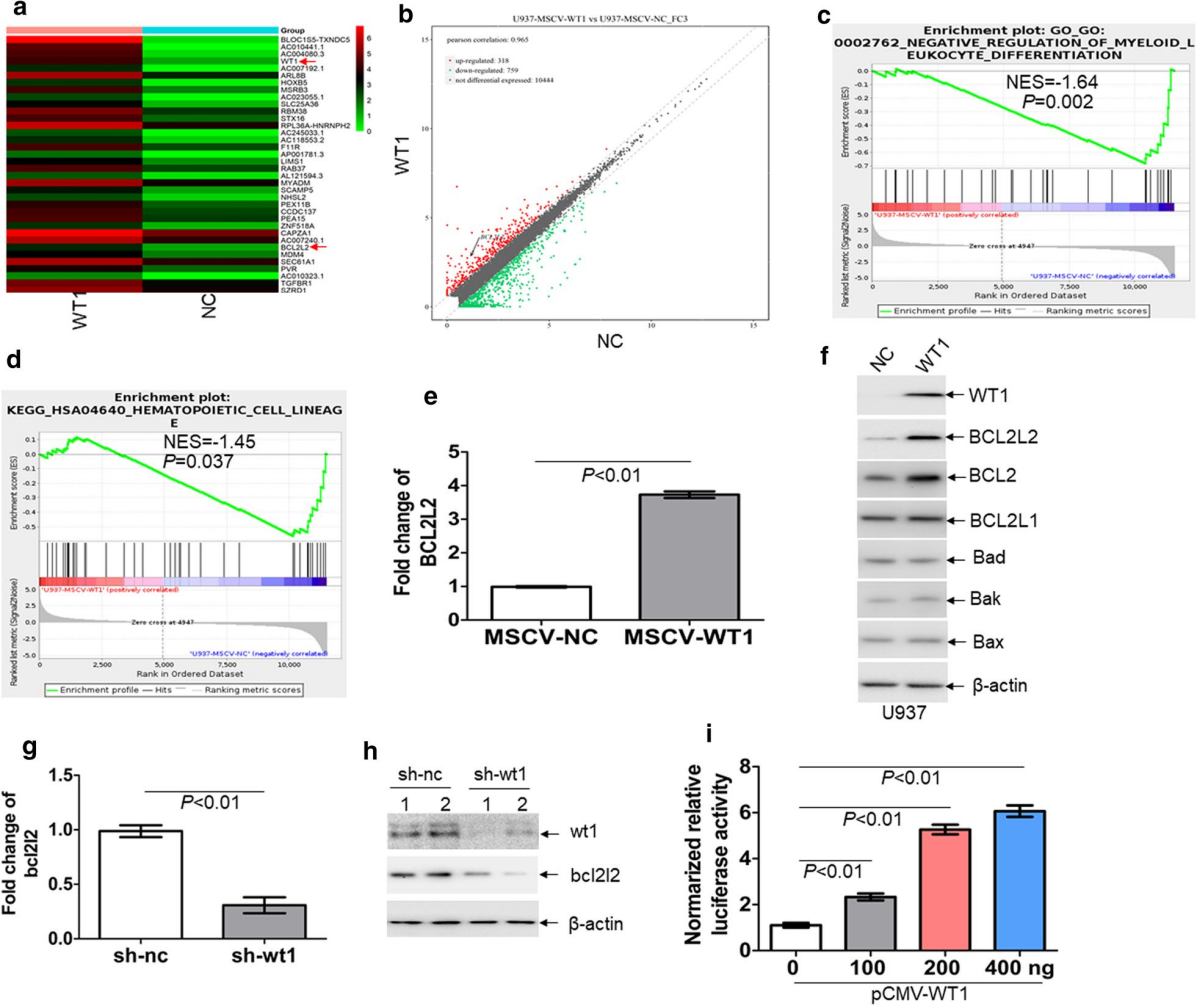
*BCL2L2* is a transcriptional target of *WT1*. RNA sequencing was performed on U937 cells transduced with retroviral MSCV-WT1 or negative control MSCV-NC vectors. **a** Heatmap representation of genes regulated by *WT1*. Shown is *BCL2L2*, which is positively regulated by *WT1*. **b** Scatter plots of genes exhibiting twofold upregulation (red plots) or twofold downregulation (green plots) upon expression of *WT1*. **c** and **d** Gene set enrichment analysis (GSEA) was performed comparing transcriptional profiles of U937 cells with overexpression of *WT1* in comparison to NC control. The enrichment score plot shown corresponds to the regulation of myeloid cell differentiation genes (**c**) and hematopoietic cell lineage genes (**d**). The NES and *P*-values are shown. **e** The transcriptional expression of *BCL2L2* was measured in U937 cells, which were transduced with MSCV-WT1 or control MSCV-NC. **f** The protein expression of BCL2, BCL2L1, BCL2L2, BAK, BAD, and BAX were determined in U937 cells transduced with MSCV-WT1 or control MSCV-NC. **g** The transcript of *bcl2l2* was measured in BM GFP^+^ blasts isolated from MLL-AF9-induced murine leukemia with knockdown of *wt1* (n = 4) and control *nc* (n = 4). **h** The protein levels of wt1 and bcl2l2 were assessed in BM GFP^+^ blasts isolated from MLL-AF9-induced murine leukemia with knockdown of *wt1* (n = 2) and control *nc* (n = 2). **i** Putative *BCL2L2* promoter, including GC-rich sequences, was constructed into the pGL3-basic vector. Both firefly and renilla luciferase activities were measured in 293T cells, which were transduced with different concentrations of pCMV-WT1, as well as pGL3 vector carrying *BCL2L2* promoter. pRL-SV40 vector containing the renilla luciferase gene was transduced into 293T cells for internal control. Histograms illustrate firefly luciferase activities normalized to renilla. Normalized luciferase activity of NC-transfected cells was arbitrarily set to 1.0
  RNA sequencing analysis indicated a significant abundance of *BCL2L2* transcript. *BCL2L2* [33], is a member of the anti-apoptotic *BCL2* family. To confirm the results from RNA sequencing analysis, we measured the transcript and protein expression of BCL2L2 in U937 cells transduced with MSCV-WT1 or negative control (MSCV-NC). As expected, ectopic overexpression of *WT1* substantially increased both transcript and protein levels of BCL2L2 in U937 cells (Fig. 2e, f). Besides, overexpression of WT1 increased the protein expression of BCL2 but did not significantly affect the protein levels of BCL2L1, BAD, BAX, and BAK (Fig. 2f). Furthermore, GFP^+^ MLL-AF9 cells were isolated from leukemic mice with knockdown of *wt1* or control *nc*. The transcript (Fig. 2g) and protein expressions (Fig. 2h) of bcl2l2 were lower in GFP^+^ MLL-AF9 cells with knockdown of *wt1* than those in GFP^+^ MLL-AF9 control *nc*.
  The presence of WT1-binding sequences, such as GGGGC and GCCCG [11, 34], was found in the putative promoter from the transcript start sequence of *BCL2L2* (Additional file 5: Fig. S2a). To assess whether WT1 binds to putative *BCL2L2* promoter, we amplified GC-rich sequences into the pGL3-basic vector. This vector was transfected into 293T cells together with different concentrations of pCMV-WT1, followed by measuring firefly and renilla luciferase activities. As shown in Fig. 2i, overexpression of WT1 significantly increased the activity of luciferase containing GC-rich sequences in a concentration-dependent manner.
3. The anti-leukemic activity of WP1130
  As a transcription factor, *WT1* is enriched in LICs and LSCs and enhances the self-renewal of LSCs. Therefore, the rapid degradation of WT1 protein through the ubiquitin–proteasome pathway could be a potential therapeutic modality for AML. The strategy by inducing the accumulation of polyubiquitinated proteins for degradation is successfully used in the treatment of multiple myeloma by lenalidomide. Lenalidomide induces the degradation of IKAROS family zinc finger 1 (IKZF1) and IKZF3 proteins [35]. WP1130 rapidly induces the accumulation of polyubiquitinated oncoproteins into juxtanuclear aggresomes for degradation [20]. Therefore, we assessed the potential anti-leukemic activity of WP1130 and its effect on WT1 protein. 50% inhibition of cell growth (IC50 value) was calculated in several leukemia cell lines treated with different concentrations of WP1130 for 24 h. As indicated in Additional file 6: Fig. S3a, IC50 values were among 1–10 μM in four leukemia cell lines and IC50 values in Kasumi-1 and THP1 cells were lower than in K562 and HL-60 cells. Therefore, Kasumi-1 and THP1 cells were selected for the following tests as they were more sensitive to WP1130 than K562 and HL-60 cells. Apoptosis was measured in Kasumi-1 and THP1 cells incubated with 5.0 μM WP1130 for 24 h. Treatment with WP1130 for 24 h increased the frequency of annexin v^+^ cells by about four–sixfold (Additional file 6: Fig. S3b). To explore the potential anti-leukemic activity of WP1130 in primary AML blasts, we measured the apoptosis in four primary AML blast samples treated with WP1130. Treatment with WP1130 induced apoptosis in all four primary AML blast samples (Additional file 6: Fig. S3c). We also determined whether WP1130 suppressed short time self-renewal ability of leukemic cells in colony formation assays. Treatment with WP1130 substantially decreased colony formation abilities in Kasumi-1 and THP1 cells (Fig. 3a). CD34^+^ cells were isolated from the same four primary AML patient samples, treated with WP1130, and assessed for their capacity to form colonies. Treatment with WP1130 reduced the capacity of AML CD34^+^ cells to form colonies (Fig. 3b). To investigate the potential effects of WP1130 on normal HSPCs, we isolated CD34^+^ cells from three UCB samples and treated them with WP1130. Our results indicated that WP1130 had little effects on the apoptosis (Fig. 3c) and colony formation capabilities of the cells isolated from three normal UCB samples (Fig. 3d).

**Fig. 3:**
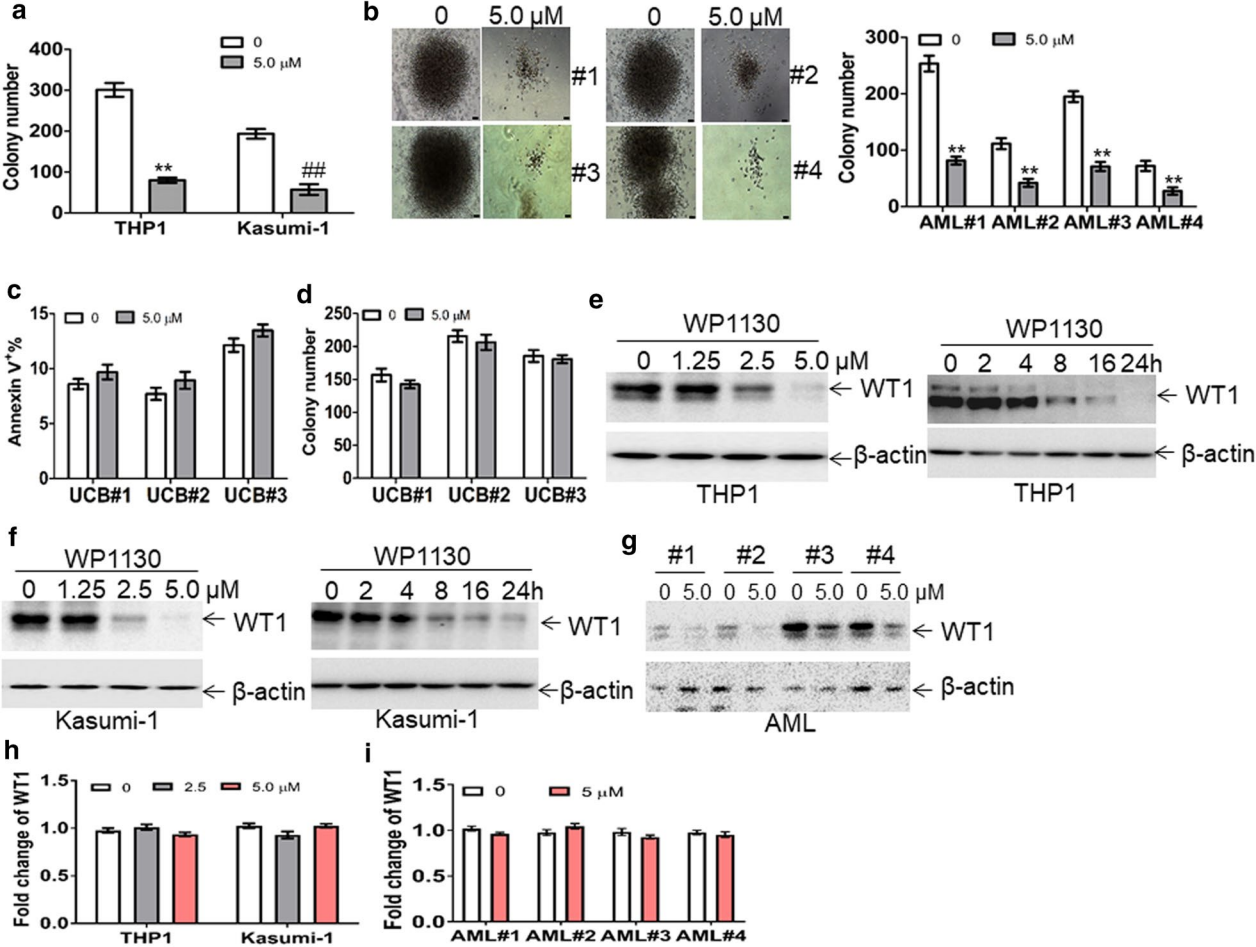
The anti-leukemic activity of WP1130 and its effect on WT1 protein. **a** THP1 and Kasumi-1 cells (2 × 10^3^) were treated with or without WP1130 (5.0 µM) and were plated on methylcellulose medium. Colonies were counted after 2 weeks. ^**^and ^##^*P *< 0.01. **b** BM CD34^+^ cells from four AML patients were isolated and incubated with or without WP1130 (5.0 µM). These cells (5 × 10^3^) were plated on methylcellulose medium, and colonies were scored after 2 weeks. ^**^P < 0.01. Shown are the representative pictures showing colonies (left) and statistical analysis of scored colonies (right). Bar represents 50 µm. **c** Apoptosis was measured in normal CD34^+^ cells isolated from umbilical cord blood and treated with or without WP1130 (5.0 µM) for 24 h. **d** Colonies formed by CD34^+^ cells (2 × 10^3^) treated with or without 5.0 μM WP1130 were scored. **e** and **f** THP1 and Kasumi-1 cells were treated with 1.25, 2.5, and 5.0 μM WP1130 for 24 h (left blots) or treated with 5.0 μM WP1130 for 2, 4, 8, 16, and 24 h (right blots). WT1 protein expression was assessed by Western blot. **g** WT1 protein levels were measured in primary blasts from four AML patients incubated with WP1130 (5.0 µM) for 24 h. **h** The transcripts of *WT1* were measured in THP1 and Kasumi-1 cells treated with 2.5 and 5.0 μM WP1130 for 24 h. **i** The transcripts of *WT1* were measured in BM blasts from four AML patients incubated with or without WP1130 (5.0 µM) for 24 h
  As WP1130 induces the degradation of target proteins by the ubiquitin–proteasome signaling pathway [20], we investigated the potential degradation of WT1 protein by WP1130 through ubiquitin–proteasome signaling pathway. THP1 and Kasumi-1 cells were treated with different concentrations of WP1130 for 24 h. Treatment with 2.5 μM concentration of WP1130 resulted in decreased levels of WT1 protein, and treatment with 5.0 μM concentration of WP1130 resulted in almost complete loss of WT1 in leukemia cell lines (Fig. 3e, f, left blots). Incubation of THP1 and Kasumi-1 cells with WP1130 for 8 h resulted in partial loss of WT1 protein, whereas incubation with WP1130 for 24 h resulted in almost complete loss of WT1 (Fig. 3e, f, right blots). To further investigate the effect of WP1130 on WT1, primary AML blasts from four AML patients were incubated with 5.0 μM WP1130. As expected, treatment with WP1130 resulted in significant loss of WT1 protein in all four primary AML blast samples (Fig. 3g). Furthermore, qRT-PCR was performed in leukemic cell lines and four primary AML blast samples to determine whether WP1130 treatment affects transcript levels of *WT1*. WP1130 did not affect mRNA expression of *WT1* in leukemic cell lines (Fig. 3h) and four primary AML blast samples (Fig. 3i).
4. WP1130 induces the rapid ubiquitination of WT1.
  As the ubiquitin–proteasome pathway affects protein expression but not mRNA expression, we then explored whether the ubiquitin–proteasome pathway mediates WP1130-induced loss of WT1 protein. WP1130-treated THP1 and Kasumi-1 cells were incubated with or without proteasome inhibitor MG132 [36]. MG132 completely blocked WP1130-induced degradation of WT1 protein (Fig. 4a), suggesting that the ubiquitin–proteasome pathway mediates WP1130-induced loss of WT1. Previous findings indicated that WP1130 could increase cellular protein ubiquitination via inhibiting deubiquitinases (DUB) activity [20]. THP1 and Kasumi-1 cells were treated with 2.5 and 5.0 μM WP1130 for 4 h, and ubiquitin was measured in whole-cell extracts. WP1130 rapidly induced a concentration-dependent accumulation of ubiquitinated proteins (Fig. 4b). To further investigate whether WP1130 specifically induced the accumulation of ubiquitinated WT1 protein, THP1 cells were treated with WP1130, followed by co-IP with anti-WT1 protein and Western blot for ubiquitin. As indicated in Fig. 4c, WP1130 dramatically increased the expression of ubiquitinated WT1 protein in THP1 cells.

**Fig. 4:**
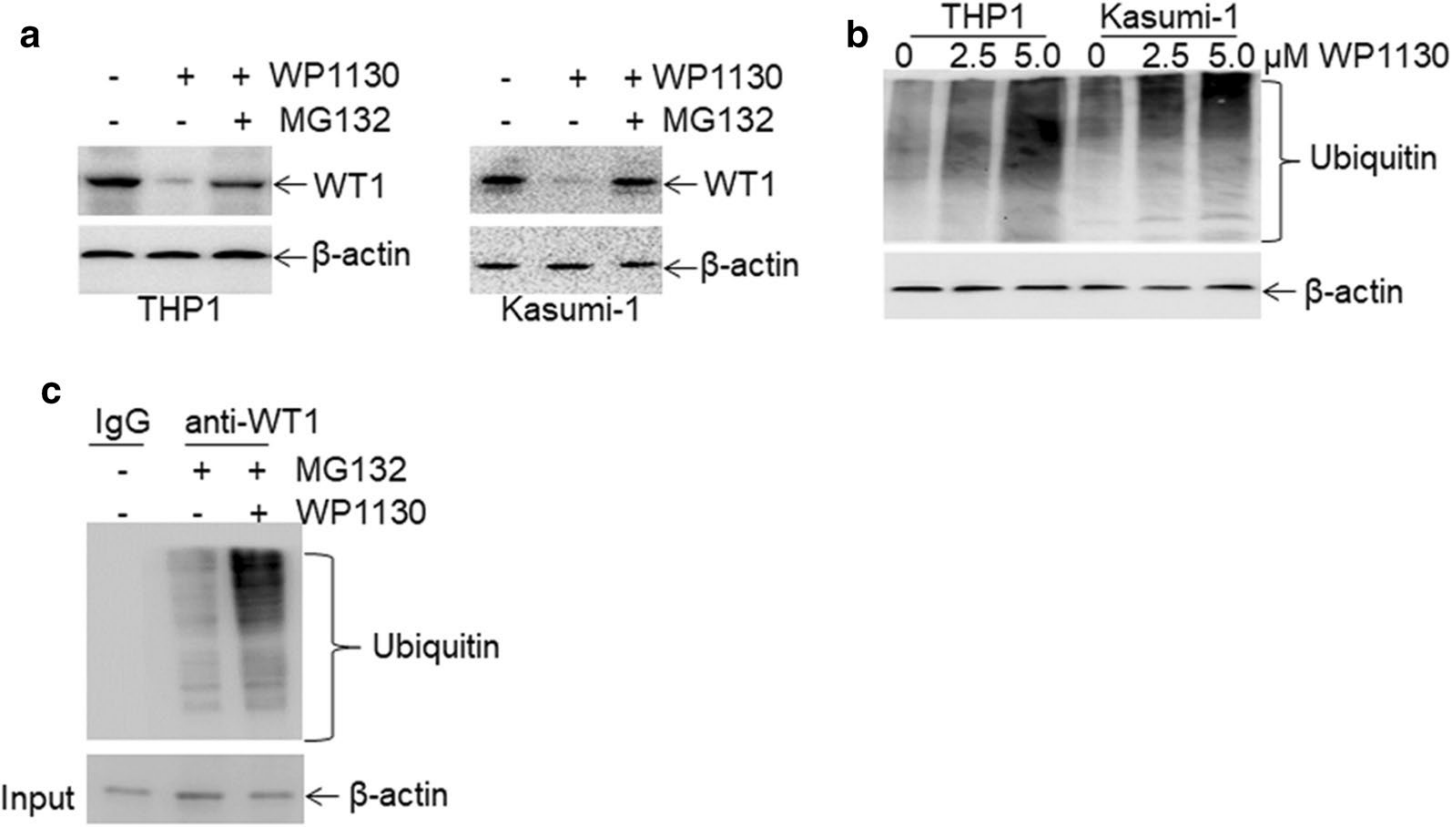
Rapid ubiquitination of WT1 protein by WP1130. **a** The protein expression of WT1 was measured in THP1 and Kasumi-1 cells treated with 5.0 μM WP1130 in the presence or absence of 5.0 μM MG132 for 24 h. **b** Ubiquitin was assessed in THP1 and Kasumi-1 cells treated with 2.5 and 5.0 μM WP1130 for 4 h. **c** THP1 cells were treated with 5.0 μM MG132 in the presence or absence of 5 μM WP1130 for 24 h, followed by co-immunoprecipitation with anti-WT1 antibody and immunoblotted for ubiquitin. The Anti-IgG antibody was used for negative control
5. Overexpression of WT1 partially prevents WP1130-induced anti-leukemic activity.
  To check the dependencies between WP1130-induced anti-leukemic activity and the degradation of WT1, we transduced leukemic cells with MSCV-WT1 or negative control vector (MSCV-NC). As indicated in Fig. 5a, the protein level of WT1 was significantly increased in THP1 and Kasumi-1 cells transduced with MSCV-WT1 in comparison to control MSCV-NC. Besides, apoptosis and colony formation assays were performed in transduced leukemic cells untreated or treated with WP1130. Overexpression of WT1 partially prevented WP1130-induced apoptosis (Fig. 5b, c) and partially blocked WP1130-induced inhibition of colony formation (Fig. 5d, e) in THP1 and Kasumi-1 cells.

**Fig. 5:**
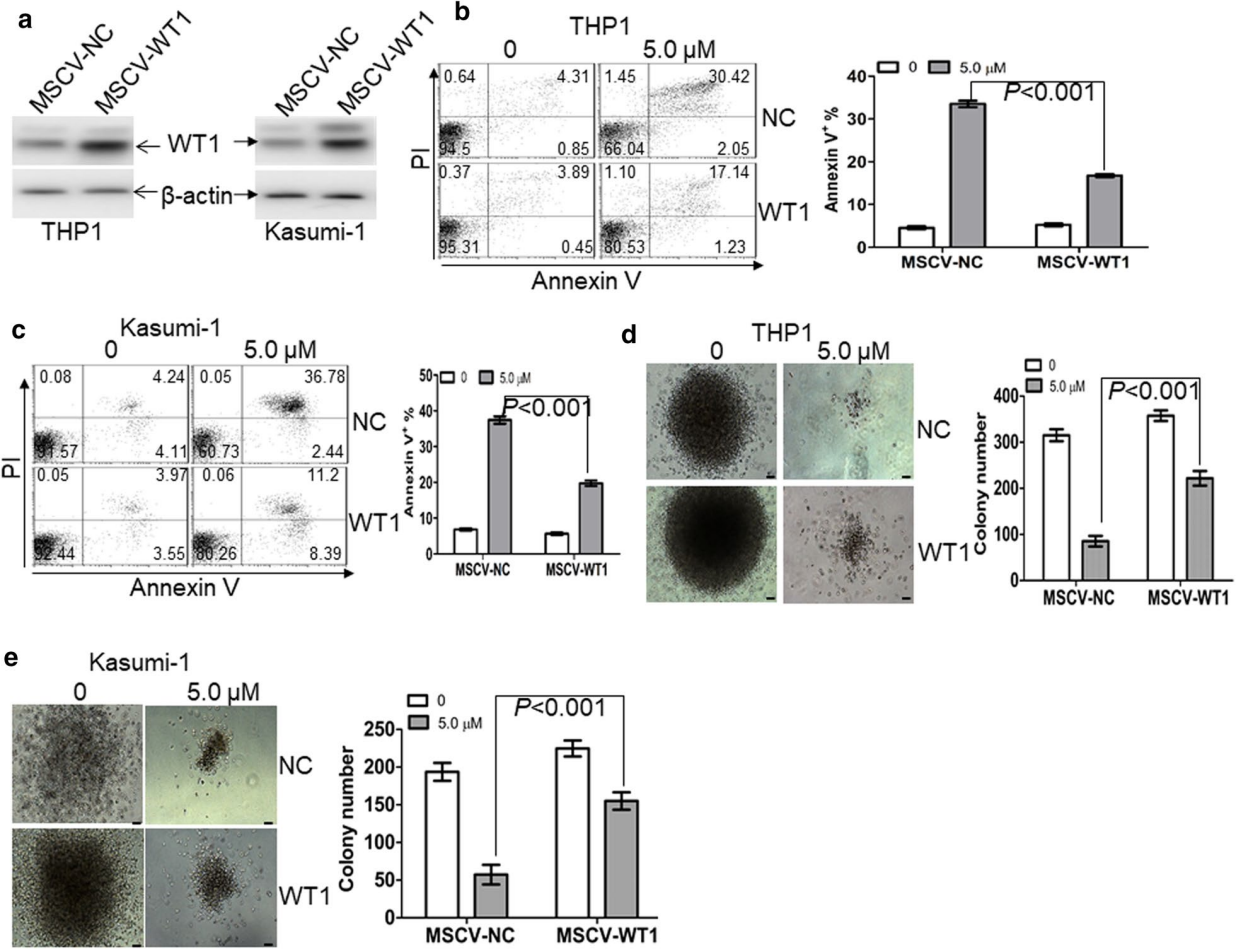
Overexpression of WT1 partially prevents WP1130-induced anti-leukemic activity. **a** The protein expression of WT1 was measured in THP1 and Kasumi-1 cells, which were transduced with control MSCV-NC or MSCV-WT1. **b** and **c** Apoptosis was measured by annexin V/PI staining in THP1 (**b**) and Kasumi-1 cells (**c**), which were transduced with control MSCV-NC or MSCV-WT1, followed by the treatment of WP1130 (5.0 µM) for 24 h. **d** and **e** Colonies were scored in THP1 and Kasumi-1 cells, which were transduced with MSCV-NC or MSCV-WT1 and then treated by WP1130 (5.0 µM) for 24 h. Shown are the representative pictures showing colonies (left) and statistical analysis of colonies scored (right). Bar represents 50 µm
6. The anti-leukemic activity of WP1130 in THP1-xenografted NSG mice.
  Because WP1130 presents vigorous anti-leukemic action in vitro, we next investigated the anti-leukemic efficacy of WP1130 in vivo using NSG mice xenografted by THP1-GFP^+^ cells. When the percentage of GFP^+^ leukemic cells in blood exceeded 5%, mice were subjected to treatment with WP1130 until vehicle-treated mice developed AML-like disease (Additional file 7: Fig. S4a). The percentage of GFP^+^ leukemic cells in the peripheral blood of WP1130-treated mice was significantly decreased in comparison to vehicle-treated mice (Additional file 7: Fig. S4b). Additionally, blood smears indicated that the percentage of leukemic blasts (THP1 cells) is lower in blood from WP1130-treated mice compared with vehicle-treated mice (Additional file 7: Fig. S4c). These results suggest that WP1130 significantly decreased the infiltration of THP1 cells in NSG mice. Finally, the overall survival time was evaluated in vehicle- and WP1130-treated mice. WP1130-treated mice showed longer survival time (median survival: 40.5 days vs. 33 days; *P* < 0.01) compared with vehicle-treated mice (Additional file 7: Fig. S4d).
7. WP1130 attenuates the self-renewal potential of LSC and prolongs the overall survival of MLL-AF9-transduced murine leukemia.
  To further assess the role of WP1130 on the self-renewal of LSC, MLL-AF9-induced murine AML model [29] was used to investigate the possible anti-LSC self-renewal activity by WP1130. GFP^+^ cells were isolated from MLL-AF9-transduced murine leukemia and then transplanted into irradiated recipient mice, followed by the treatment with vehicle control or WP1130 (Fig. 6a). The frequency of GFP^+^ cells was measured in BM cells when vehicle mice developed full-onset leukemia. GFP^+^ cells were decreased by about threefold in WP1130-treated mice in comparison to vehicle-treated mice (Fig. 6b). Moreover, blood and BM smears indicated a substantial decrease of leukemic blasts in WP1130-treated mice in comparison to vehicle-treated mice (Fig. 6c). Accordingly, WP1130 reduced spleen weight by almost twofold (Fig. 6d). To determine the effect of WP1130 on the frequency of LICs, we measured the percentage of c-Kit^+^Mac-1^+^ cells in BM GFP^+^ cells. The frequency of LICs was decreased by threefold in WP1130-treated mice in comparison to vehicle-treated mice (Fig. 6e). Furthermore, limiting dilution assay was performed to assess the frequency of LSC in WP1130- and vehicle-treated mice. WP1130 treatment led to a 67% decrease in the frequency of LSCs (Fig. 6f and Additional file 9: Table S4). BM GFP^+^ cells from leukemic mice were sorted, and colony formation assay was performed. Treatment with WP1130 decreased the colony formation ability of GFP^+^ leukemic cells (Fig. 6g).

**Fig. 6:**
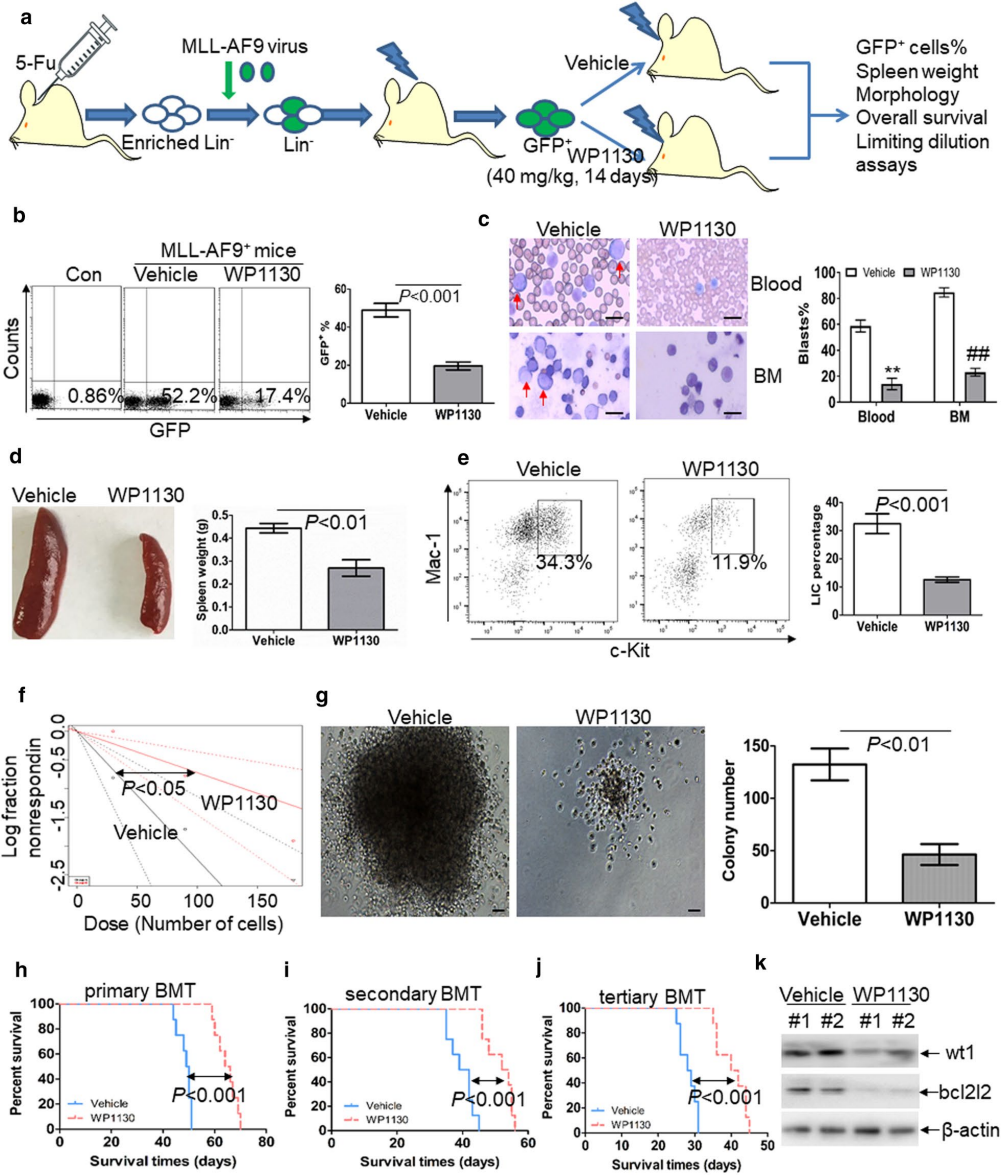
Anti-self-renewal effect of WP1130 on LSCs in MLL-AF9-induced murine leukemia. **a** A schematic outline of the in vivo experiment using MLL-AF9-induced murine leukemia model untreated or treated with WP1130. **b** GFP^+^ cells were measured in BM cells isolated from MLL-AF9-induced leukemic mice untreated (n = 4) or treated with WP1130 (n = 4) upon the development of full-onset leukemia by vehicle-treated mice. Shown are the representative plots (left) and statistical analysis of the frequency of GFP^+^ cells (right). **c** A representative image of blood and BM smear of vehicle- or WP1130-treated MLL-AF9 mice (left) and statistical analysis of the average percentage of leukemic blasts in blood and BM (right). Bar represents 10 µm, and these images were amplified 200 folds. ^**^ and ^##^*P *< 0.01. **d** A representative image of the spleen (left) and statistical analysis of spleen weight (right) from the vehicle-(n = 4) or WP1130-treated mice (n = 4). **e** The frequencies of LICs in GFP^+^ cells were assessed in BM cells from vehicle- (n = 4) or WP1130-treated mice (n = 4). Shown are the representative plots (left) and statistical analysis of LICs (right). **f** Limiting dilution assay of BM GFP^+^ cells from vehicle- (n = 6) or WP130-treated mice (n = 6). The frequency of LSCs and the *P*-value were calculated by L-calc software. **g** BM GFP^+^ cells were isolated from vehicle- (n = 4) or WP1130-treated mice (n = 4) and then plated on methylcellulose medium. Colonies were scored after 2 weeks. Shown are the representative pictures showing colonies (left) and statistical analysis of the number of colonies (right). Bar represents 50 µm. (**h**–**j**) Overall survival was analyzed in the primary BMT (**h**, n = 8), secondary BMT (**i**, n = 8), and tertiary BMT (**j**, n = 8) of MLL-AF9-induced leukemia mice treated with vehicle or WP1130. (K) Wt1 and bcl2l2 protein expressions were measured in BM cells from vehicle- (n = 2) or WP1130-treated mice (n = 2)

Serial murine BMT tests were performed to explore whether WP1130 treatment inhibits the long-term self-renewal of LSCs. WP1130 treatment significantly prolonged the overall survival in primary MLL-AF9-transduced leukemia mice (Fig. 6h, median survival of WP1130 versus vehicle, 65 days versus 49.5 days, respectively; *P *< 0.001). In the secondary BMT assay, GFP^+^ cells from BM of vehicle- or WP1130-treated mice were transplanted to lethally irradiated recipient mice. The overall survival time was significantly longer in WP1130-treated mice than in vehicle-treated mice (Fig. 6i, median survival time of WP1130 versus vehicle, 53 days versus 40.5 days, respectively; *P *< 0.001). Then, tertiary mouse BMT was performed using secondary leukemia BM blasts as donor cells. The overall survival time was significantly prolonged in WP1130-treated mice than in vehicle-treated mice (Fig. 6j, median survival of WP1130 versus vehicle, 41 days versus 28.5 days, respectively; *P *< 0.001).

Considering the high conservation of WT1 amino acids sequence (greater than 95%) between human and mouse [37], we then assessed whether WP1130 also degrades murine wt1 protein in vivo. Murine wt1 protein expression was measured in GFP^+^ AML cells from vehicle- and WP1130-treated mice. As expected, treatment with WP1130 resulted in the loss of murine wt1 (Fig. 6k), as well as the bcl2l2 protein (Fig. 6k).

## Discussion

**Fig. 7:**
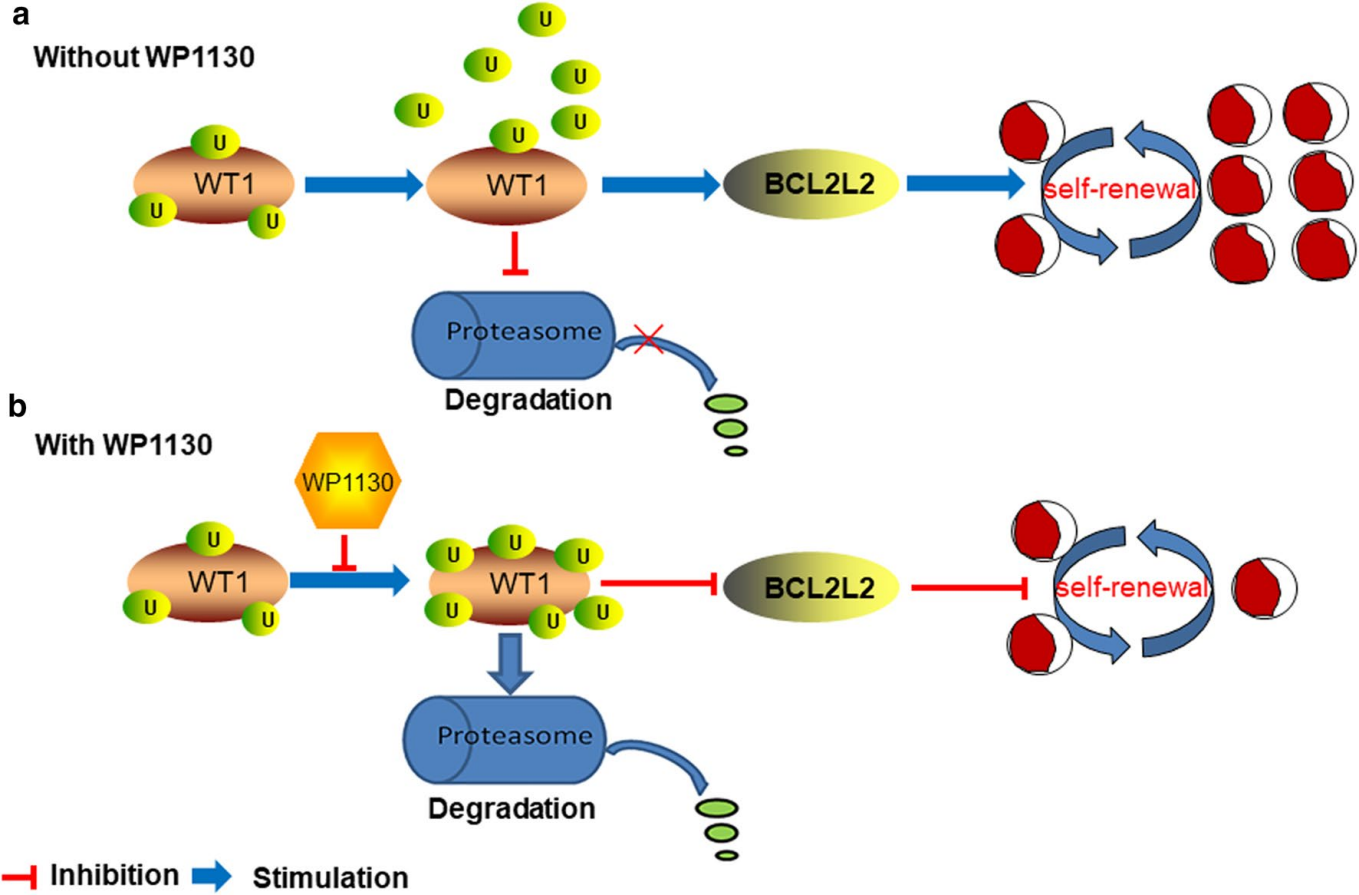
An illustration of the anti-self-renewal activity of WP1130 and its effect on WT1 protein. **a** Overexpression of WT1 in leukemia cells contributes to the proliferation and self-renewal of LIC by positively regulating BCL2L2 expression. **b** WP1130 treatment enhances the ubiquitination of WT1 protein, leading to its degradation via the ubiquitin–proteasome pathway in leukemic cells. The downregulation of WT1 reduces the proliferation and self-renewal of LIC via inhibition of BCL2L2 expression

In this study, we investigated the role of *WT1* in the maintenance of AML and the anti-leukemic ability of WP1130. Our results suggest that WT1 facilitates the proliferation and self-renewal of LSCs by positive regulation of BCL2L2 expression (Fig. 7a). Our data also suggest that treatment with deubiquitinase inhibitor WP1130 rapidly degrades WT1 protein via ubiquitin–proteasome signaling. The downregulation of WT1 inhibits the proliferation and self-renewal of LSCs by inhibiting BCL2L2 expression (Fig. 7b). Therefore, WT1 is required for the development of AML, and treatment with WP1130 might represent a useful therapeutic modality for AML patients by affecting the WT1-BCL2L2 axis.

Targeting WT1 is a promising therapeutic strategy in solid tumors and hematological malignancies because *WT1* is necessary for tumor growth and leukemogenesis. Inhibition of *WT1* will decrease proliferation and induce apoptosis by affecting important genes [38]. Here, we report that *BCL2L2* is a new transcriptional target of *WT1*. *BCL2L2* plays an important role in regulating cell survival and death [39]. Targeting *BCL2L2* by shRNA induced apoptosis in lymphoma cell lines and higher levels of *BCL2L2* in patients with B lymphocyte leukemia are associated with lower overall survival [40]. Furthermore, *BCL2L2* is significantly overexpressed in aggressive and indolent lymphomas [33]. The knockdown of *WT1* induces cell growth arrest and apoptosis through modulating BCL2 expression [15, 38]. Consistent with previous studies, our results also indicate that overexpression of WT1 increases the expression of BCL2 (Fig. 2f). *BCL2*, as an important anti-apoptotic molecule, can control cell proliferation and self-renewal of LSCs. Inhibition of BCL2 decreases oxidative phosphorylation and selectively eradicates quiescent LSC [41]. Venetoclax, a specific BCL2 inhibitor approved by the Food and Drug Administration (FDA), demonstrates strong anti-leukemic activity and improves the overall survival in elderly AML patients [42, 43]. Our data suggest that WP1130 may present anti-self-renewal activity by modulating the WT1-BCL2L2/BCL2 axis in leukemic cells. WP1130 is a potent inhibitor for this axis, and combined treatment with Venetoclax might exhibit stronger anti-leukemic potential than single Venetoclax or WP1130 treatment.

WP1130 is a small molecular compound that strongly inhibits the activity of deubiquitinating enzymes [44]. WP1130 treatment leads to the degradation of proteins by inducing the accumulation of protein-ubiquitin conjugates [21]. For example, WP1130 induces the degradation of P53 protein through accumulating P53-ubiquitin conjugates in hepatocellular carcinoma and non-small cell lung carcinomas [22, 45]. Consistent with these reports, our results also indicate that WP1130 induces the destruction of WT1 protein through accumulating WT1-ubiquitin conjugates. WP1130 induces the degradation of WT1 in a shorter time and lower concentration, by contrast with curcumin, a natural flavonoid from the rhizome of *Curcuma longa,* which inhibits the expression of WT1 through PKCα signaling pathway [16]. Accumulation of protein-ubiquitin conjugates for degradation is a promising therapeutic strategy to eradicate some undruggable proteins [46]. Therefore, WP1130 is a promising agent for treatment with AML patients through the degradation of WT1 protein.

LSC is characterized by their unlimited self-renewal potential and has a pivotal role in the relapse and refractory of AML [47]. Thus, elucidating the molecular mechanism, which underlies the unlimited self-renewal ability of LSC, might facilitate the development of LSC-targeted therapy [1]. Several genes play an important role in the self-renewal of LSC. For example, *cyclin*-*dependent kinase 6* (*CDK6*) [48] and *jumonji domain*-*containing 1C* (*JMJD1C*) [49] are required in MLL-AF9-induced murine leukemia. Although overexpression of *WT1* facilitates the proliferation of leukemic cells, it is still unknown whether *WT1* facilitates the self-renewal of LSC. Our results indicate that knockdown of *wt1* substantially decreases the frequency of LSC, impairs LSC self-renewal ability, and prolongs the overall survival in MLL-AF9-induced murine leukemia, suggesting that *WT1* is required for the development of leukemia. Therefore, our data indicate that WT1 may be indispensable for the stemness of LSC and may facilitate the development of AML.

LSC is responsible for chemotherapy resistance and relapse in AML patients and acts as a key therapeutic target for AML [1]. LSC drives both disease progression and relapse through unlimited self-renewal, loss of differentiation, and drug resistance. Therefore, eradicating LSC by molecular compounds provides a successful therapeutic strategy. WP1130 decreases not only short term self-renewal of LSC but also extends overall survival time in serial BMT assay, which measures long term self-renewal capacity of LSC. Besides, WP1130 decreases the frequency of LSC as assessed by limiting dilution assay. Thus, we speculate that WP1130 treatment might prolong survival time by decreasing the frequency of LSC. Previously published studies indicate that knockdown of *WT1* decreases the colony formation capacity in human leukemic cells [18, 50]. Most importantly, our data show that knockdown of *wt1* decreases the colony formation, inhibits the frequency of LSC, and extends the overall survival in MLL-AF9-induced murine leukemia, indicating that knockdown of *wt1* suppresses the self-renewal of LSC. Therefore, we speculate that WP1130 inhibits the self-renewal ability of LSC by degrading WT1 protein, leading to prolonged survival.

## Conclusions

Here, we find that *wt1* is overexpressed in LICs and LSCs. WT1 facilitates the self-renewal of LSCs and is required for the maintenance of AML by increasing the expression of BCL2 and BCL2L2. WP1130 exhibits strong anti-leukemic activity by degrading WT1 protein. More importantly, WP1130 decreases the frequency of LSCs and extends the overall survival in THP1-xenografted NSG and MLL-AF9-induced murine AML models. Thus, we propose that WP1130 may be a potential anti-LSCs compound for AML patients with high expression of WT1.

## Supplementary information

**Additional file 1: Table S1.** Clinical characteristics of AML patients.

**Additional file 2:** Materials and methods.

**Additional file 3: Fig. S1** Knockdown of *wt1* inhibits the self-renewal of LSC in MLL-AF9-induced murine leukemia. **a** MLL-AF9-induced murine leukemia blasts were transduced with shRNA for *wt1* (sh-wt1) or control *nc*, and were transplanted into receipt mice. The transcript of *wt1* was measured in BM mononuclear cells from recipient mice xenografted with MLL-AF9-induced leukemia with sh-wt1 (n = 4) or control *nc* (n = 4) at the endpoint. **b** Wright-Giemsa staining for the blood and BM blasts from recipient mice xenografted with MLL-AF9-induced leukemia with knockdown of *wt1* or control *nc* (left). Arrows indicate leukemic blasts. Bar represents 10 µm, and these images were amplified 200 fold. More than 100 nuclear cells were counted to obtain blast percentage in blood and BM (right).

**Additional file 4: Table S2.** The sequences of primers for qRT-PCR and construction of plasmids.

**Additional file 5: Fig. S2 a** Indication of putative *BCL2L2* promoter sequence for *WT1*.

**Additional file 6: Fig. S3** The potential anti-leukemic activity of WP1130 in leukemic cells. **a** Four leukemic cell lines were treated with different concentrations of WP1130 for 24 h. Cell growth was assessed by CCK-8 assay. A 50% inhibitory concentration (IC50) of WP1130 was calculated for the four cell lines. **b** Apoptosis was measured by Annexin V/PI staining in THP1 and Kasumi-1 cells, which were treated with 5.0 μM WP1130 for 24 h. ^**^and ^##^*P* < 0.01 versus untreated cells. Shown are the representative plots (left) and statistical analysis of Annexin V^+^ cells. **c** Apoptosis was measured in four primary AML blasts treated with or without WP1130 for 24 h. ^**^P < 0.01 versus untreated cells.

**Additional file 7: Fig. S4** Anti-leukemia activity of WP1130 in THP1-GFP-xenografted NSG mice. **a** A schematic outline of the *in vivo* experiment using THP1-GFP-xenografted NSG mice treated with WP1130 or not. **b** GFP^+^ cells were measured in peripheral blood from vehicle mice (n = 4) or WP1130-treated mice (n = 4) when the vehicle mice became moribund after engraftment. Shown are the representative plots (left) and statistical analysis of GFP^+^ cells (right). **c** The representative images of blood smear were shown by Wright-Giemsa’s stain when the vehicle mice became moribund (left) and statistical analysis of the percentage of leukemia blasts in the blood (right). Bar represents 10 µm, and these images were amplified 200 fold. **d** Overall survival was indicated in THP1-GFP-xenografted NSG mice treated with (n = 6) or without WP1130 (n = 6).

**Additional file 8: Table S3.** Limiting dilution assay of MLL-AF9-induced mouse leukemia transduced with sh-nc or sh-wt1.

**Additional file 9: Table S4.** Limiting dilution assay of MLL-AF9-induced mouse leukemia treated with or without WP1130.

## Data Availability

The datasets used and/or analyzed during the current study are available from the corresponding author on reasonable request

## Abbreviations

WT1: Wilm’s tumor-1
shRNAs: Hairpin RNAs
USP: Ubiquitin-specific protease
LIC: Leukemia-initiating cell
LSC: Leukemia stem cell
HSPC: Hematopoietic stem and progenitor cell
AML: Acute myeloid leukemia
DUB: Deubiquitinating enzymes
ELDA: Extreme limiting dilution assay
qRT-PCR: Quantitative real-time PCR
NSG: NOD/SCID-IL2Rγ mice
BM: Bone marrow
5-FU: 5-fluorouracil
OS: Overall survival
BMT: Bone marrow transplantation
IC50 value: 50% inhibition of cell growth
NSG: NOD/SCID-IL2Rγ
BCL2: B cell lymphoma-2
UCB: Umbilical cord blood
SCF: stem cell factor
Flt3l: Fms related receptor tyrosine kinase 3 ligand
IL-3: Interleukin-3
IL-6: Interleukin-6
TPO: Thrombopoietin
GFP: Green fluorescent protein
DMSO: Dimethyl sulfoxide
PEG300: Polyethylene glycol 300
