# Supplementary material for: WT1 facilitates the self-renewal of leukemia-initiating cells through the upregulation of BCL2L2: WT1-BCL2L2 axis as a new acute myeloid leukemia therapy target
Source: J Transl Med. 2020 Jun 24;18:254. doi: 10.1186/s12967-020-02384-y (PMC7313134; doi:10.1186/s12967-020-02384-y)
Supplement: Supplementary file 1 — Additional file 1: Table S1. Clinical characteristics of AML patients. [file 12967_2020_2384_MOESM1_ESM.docx]

**Table S1. Clinical characteristics of AML patients**

| Num | Sex | Age (y) | FAB subtype | Karyotype | Blasts % |
| --- | --- | --- | --- | --- | --- |
| #1 | F | 58 | M2 | 46, XX+t (8;21) | 76 |
| #2 | M | 55 | M4 | 46, XY | 82 |
| #3 | M | 46 | M5 | 47, XY, +13 | 72 |
| #4 | M | 49 | M5 | 46, XY | 75 |

All bone marrow samples were obtained from untreated AML patients
