# Supplementary material for: WT1 facilitates the self-renewal of leukemia-initiating cells through the upregulation of BCL2L2: WT1-BCL2L2 axis as a new acute myeloid leukemia therapy target
Source: J Transl Med. 2020 Jun 24;18:254. doi: 10.1186/s12967-020-02384-y (PMC7313134; doi:10.1186/s12967-020-02384-y)
Supplement: Supplementary file 2 — Additional file 2: Materials and methods. [file 12967_2020_2384_MOESM2_ESM.docx]

**Supplementary materials and methods**

**Luciferase detection**

For the luciferase reporter assay, 293T cells were transfected with different concentrations of pCMV-WT1 and pGL-3-BCL2L2 by Lipofectamine^TM^ 2000 (Life Technologies, Carlsbad, CA, USA) according to the manufacturer's instruction. Cells were also co-transfected with the pRL-TK control vector (Promega, Madison, WI, USA) encoding Renilla luciferase for normalizing transfection efficiency. The luciferase reporter assay was performed in 293T cells using the Dual-Luciferase^®^Reporter Assay System (Promega, Madison, WI, USA) at 48 h after transfection. The results were calculated as the relative luciferase unit (RLU) by calculating the ratio of Firefly luciferase to the Renilla luciferase.

**Western blot**

Western blot analysis was performed according to standard procedure. Briefly, total protein lysates were extracted from cells by lysing the samples in cold RIPA buffer with sodium orthovanadate, phenylmethylsulfonyl fluoride, and protease and phosphatase inhibitors (Thermo Scientific, Waltham, MA, USA). Protein concentration was evaluated by a BCA assay (Thermo Scientific). The following antibodies were used: WT1 (Abcam, Cambridge, MA, USA); BCL2L2 (ab190952, Abcam); BCL2 (ab59348, Abcam); BCL2L1 (ab32370, Abcam); BAK (ab32371, Abcam); BAX (ab32503, Abcam); BAD (ab32445, Abcam); ubiquitin (sc-8017, Santa Cruz Biotechnology, Santa Cruz, CA, USA); β-actin antibody (ab6276, Abcam) as an internal control. All primary antibodies were diluted in 1×TBST buffer. All secondary antibod­ies are conjugated with horseradish peroxidase (HRP). Signals were detected by chemiluminescence reagents (Thermo Scientific).

**Apoptosis assay**

Leukemic cells were plated at 2×10^5^ cells/ml in a six-well plate. Apoptosis was measured by annexin V/Propidium Iodide (PI, Invitrogen) staining. Briefly, cells were collected and washed by 1×binding buffer and incubated in working solution (100 μl 1×binding buffer with 2.5 μl annexin V-FITC and 2.5 μl PI) for 15 min. Cells were resuspended with 400 μl 1×binding buffer and analyzed by flow cytometry (Becton Dickinson, Mountain View, CA, USA) within 30 min after staining.

**Cell proliferation by CCK8**

Leukemic cells (1×10^5^ cells/ml) were seeded in 96-well plates and were treated with or without different concentrations of WP1130 for 24 h. Then, CCK-8 solution (10 μl, Dojindo, Kumamoto, Japan) was added in 96-well plates and incubated for four hours. The absorbance was measured at 450 nm by using an MRX II microplate reader (Dynex, Chantilly, VA, USA).

**Construction of plasmids**

The whole CDS of *WT1* isoform (Ex5-/KTS-; NM_000378) were directly synthesized (Genewiz, Suzhou, China) and then constructed into retroviral pMSCV-puro (Clontech) and pCMV vectors (Stratagene, La Jolla, CA, USA), respectively. The promoter sequence of *BCL2L2* was amplified and inserted in a pGL-3 plasmid. Gene-specific short hairpin RNA (shRNA) for murine *wt1* was designed and cloned into pSIREN-RetroQ (Clontech) retroviral vector. Control shRNA is a nonfunctional construct. All of the primer sequences were shown in Table S4 and all of these constructs were confirmed by sequencing.

**Retroviral production and cell transduction**

HEK293T cells (4×10^6^) were plated in 10 cm dishes. After 24 h, all constructed plasmids together with negative control vectors were co-transfected with packaging plasmids into HEK293T cells. The virus was harvested from the supernatant at 48 h after transfection, and further was filtered through a 0.45 μm low protein-binding-polysulfone filter (Millipore, Billerica, MA, USA). Leukemic cells (2×10^5^/ml) were suspended in viral supernatant with 8 μg/ml polybrene (Sigma-Aldrich, St. Louis, MO, USA) and centrifuged at 2000×rpm for 2 h. Puromycin (2 μg/ml, Medchemexpress, Princeton, NJ, USA) was added into the supernatant to select positive clones.

**Co-****immunoprecipitation (co-IP) assays**

Cell lysates were immunoprecipitated by anti-WT1 antibody (1:200) using the Pierce™ co-immunoprecipitation kit (#88804, Thermo Scientific) according to the manufacturer's protocol. Briefly, leukemic cells were lysed in cold RIPA lysis buffer. The supernatant was transferred to new tubes after centrifugation, and protein A/G agarose was added to eliminate non-specific binding proteins. The primary antibody was added in the tube, and the antigen/antibody complex was slowly rotated on the rotating shaker at 4°C overnight. The beads were washed with IP lysis, and the antigen/antibody complex was bound to protein A/G magnetic beads for 1 hour. The antigen/antibody complex was washed and eluted for the following Western blot.

**Flow cytometry analysis**

BM cells from transplanted mice were harvested and stained at 4℃ with various antibodies diluted in flow cytometry staining buffer for 30 minutes for staining of primary antibody. Antibodies used for flow cytometry include Streptavidin-APC-R700; c-Kit-PE; Sca-1-PE-Cy7; CD34-APC; CD16/CD32-PerCP-Cy5.5; Mac-1-PE-Cy7 (all from BD PharMingen, San Diego, CA, USA). The frequencies of GMP-like leukemic cells (L-GMP, GFP^+^Lin^-^c-Kit^+^Sca-1^-^CD34^+^CD16/32^+^) as LSCs and c-Kit^+^Mac-1^+^ cells as LICs from MLL-AF9-induced murine leukemia were measured by flow cytometry. Analysis and cell sorting were performed by FACS Vantage or FACS Aria II (Becton Dickinson), respectively. Data were analyzed with FlowJo software v10.0 (Ashland, OR, USA).

**Colony-forming assays**

Bone marrow mononuclear cells (blasts%>70%) from AML patients were separated by Ficoll-Paque liquid and suspended in IMDM (Gibco). Normal CD34^+^ cells were isolated from umbilical cord blood (UCB) by immunomagnetic positive selection kit (Stemcell Technologies). AML blasts, leukemic cell lines, and normal CD34^+^ cells were seeded into methylcellulose medium (MethoCult^TM^ H4434 Classic, Stemcell Technologies) for ten days. BM GFP^+^ cells from MLL-AF9-induced murine leukemia were isolated and plated into methylcellulose medium (MethoCult™ GF M3434, Stemcell Technologies). Colonies (>40 cells) were counted according to the manufacturer's protocol.

**RNA sequencing analysis**

Total RNA was extracted by using Trizol reagent (Invitrogen, Carlsbad) according to the manufacturer’s protocol. RNA concentration and quality were measured by DS-11 spectrophotometer (DeNovix). KAPA Stranded RNA-Seq Library Preparation Kit (Illumina Technologies, San Diego, CA, USA) was used to construct the cDNA library as the following: poly-A mRNA was isolated from total RNA by using the NEB-Next Oligo d(T) magnetic beads. Then, mRNA was fragmented into small pieces after treatment with the fragmentation buffer. cDNA was synthesized by random primers and DNA polymerase I. Final cDNA library was constructed through suitable fragments by PCR amplification. Sequencing was performed on Illumina HiSeq 4000 sequencing platform after double-stranded cDNA samples were verified with an Agilent 2100 Bioanalyzer (Agilent Technologies, Santa Clara, CA, USA). Image analysis, base calling, and error estimation were carried out by Illumina/Solexa Pipeline. The trimmed reads mapped to the corresponding reference genome by HISAT2 (version 2.0.4) and StringTie (version 1.2.3) were used to reconstruct the transcriptome.
