# Supplementary figures and images for: WT1 facilitates the self-renewal of leukemia-initiating cells through the upregulation of BCL2L2: WT1-BCL2L2 axis as a new acute myeloid leukemia therapy target
Source: J Transl Med. 2020 Jun 24;18:254. doi: 10.1186/s12967-020-02384-y (PMC7313134; doi:10.1186/s12967-020-02384-y)

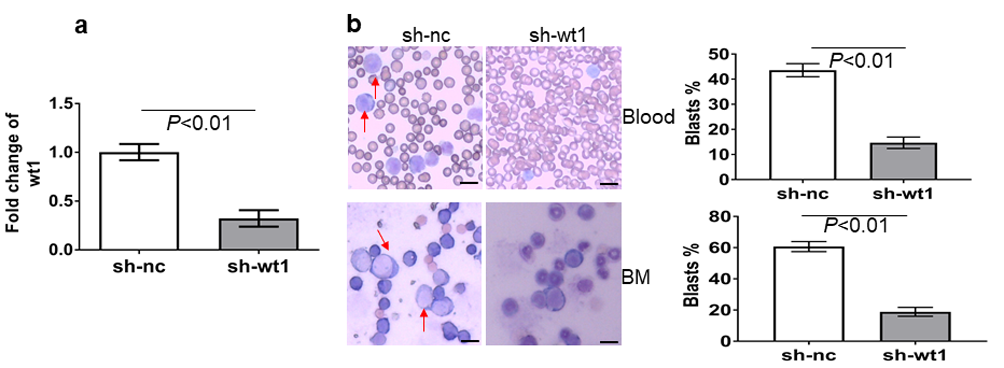

Supplement: Supplementary file 3 — Additional file 3: Fig. S1 Knockdown of wt1 inhibits the self-renewal of LSC in MLL-AF9-induced murine leukemia. a MLL-AF9-induced murine leukemia blasts were transduced with shRNA for wt1 (sh-wt1) or control nc, and were transplanted into receipt mice. The transcript of wt1 was measured in BM mononuclear cells from recipient mice xenografted with MLL-AF9-induced leukemia with sh-wt1 (n = 4) or control nc (n = 4) at the endpoint. b Wright-Giemsa staining for the blood and BM blasts from recipient mice xenografted with MLL-AF9-induced leukemia with knockdown of wt1 or control nc (left). Arrows indicate leukemic blasts. Bar represents 10 µm, and these images were amplified 200 fold. More than 100 nuclear cells were counted to obtain blast percentage in blood and BM (right). [file 12967_2020_2384_MOESM3_ESM.tif]

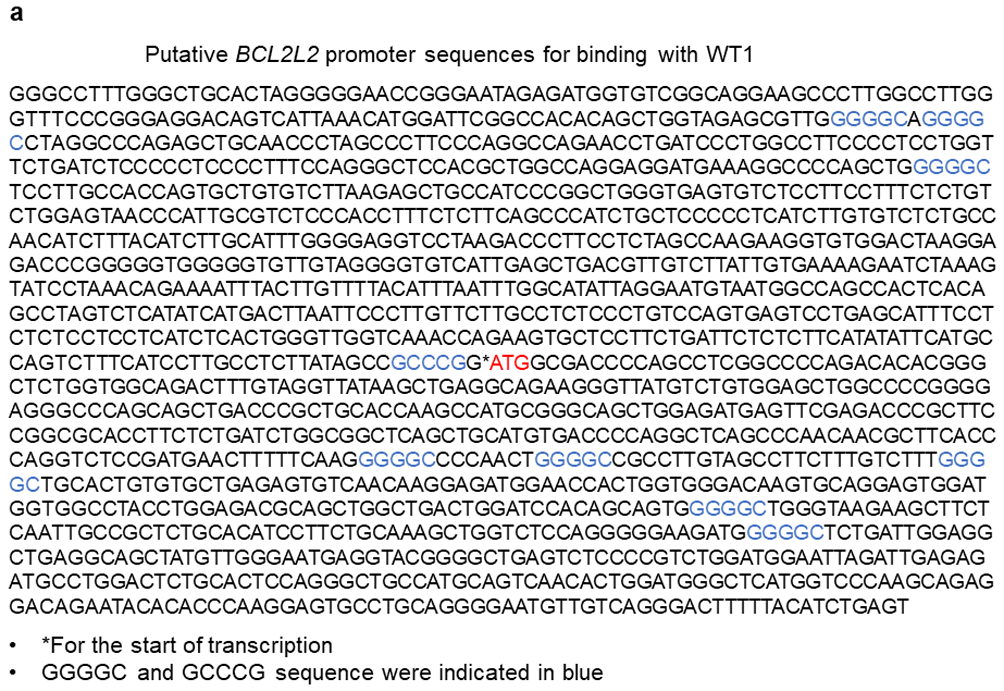

Supplement: Supplementary file 5 — Additional file 5: Fig. S2 a Indication of putative BCL2L2 promoter sequence for WT1. [file 12967_2020_2384_MOESM5_ESM.tif]

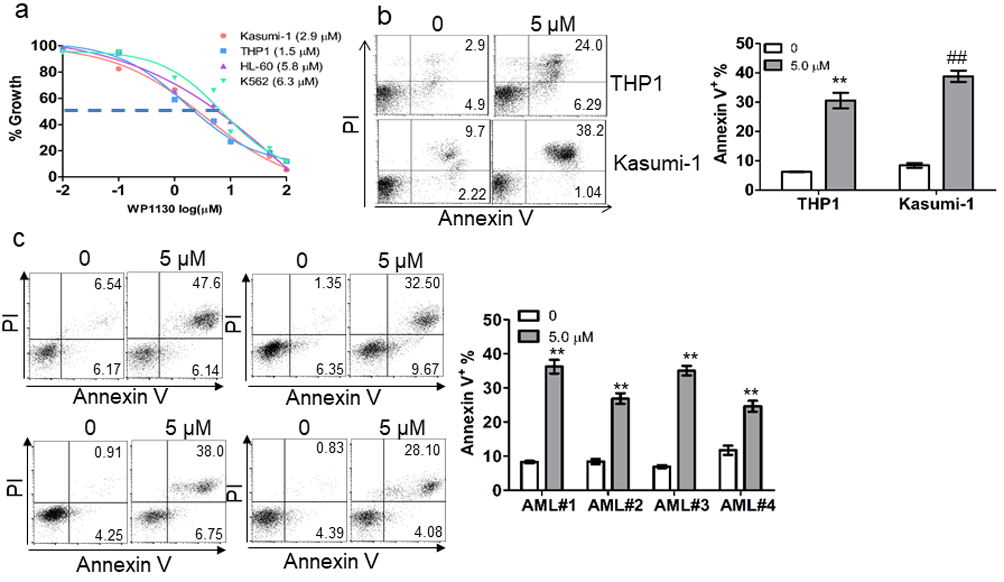

Supplement: Supplementary file 6 — Additional file 6: Fig. S3 The potential anti-leukemic activity of WP1130 in leukemic cells. a Four leukemic cell lines were treated with different concentrations of WP1130 for 24 h. Cell growth was assessed by CCK-8 assay. A 50% inhibitory concentration (IC50) of WP1130 was calculated for the four cell lines. b Apoptosis was measured by Annexin V/PI staining in THP1 and Kasumi-1 cells, which were treated with 5.0 μM WP1130 for 24 h. **and ##P < 0.01 versus untreated cells. Shown are the representative plots (left) and statistical analysis of Annexin V+ cells. c Apoptosis was measured in four primary AML blasts treated with or without WP1130 for 24 h. **P < 0.01 versus untreated cells. [file 12967_2020_2384_MOESM6_ESM.tif]

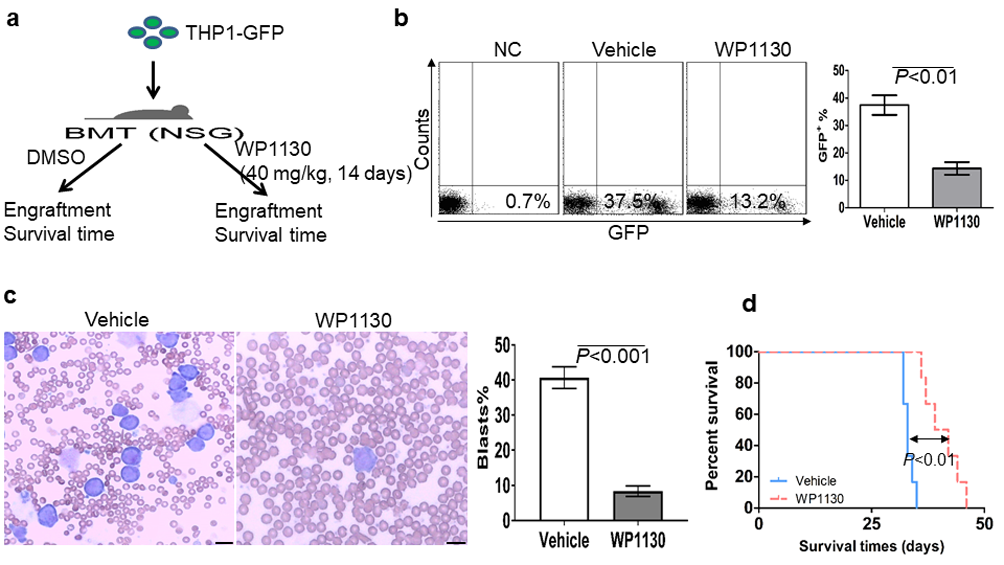

Supplement: Supplementary file 7 — Additional file 7: Fig. S4 Anti-leukemia activity of WP1130 in THP1-GFP-xenografted NSG mice. a A schematic outline of the in vivo experiment using THP1-GFP-xenografted NSG mice treated with WP1130 or not. b GFP+ cells were measured in peripheral blood from vehicle mice (n = 4) or WP1130-treated mice (n = 4) when the vehicle mice became moribund after engraftment. Shown are the representative plots (left) and statistical analysis of GFP+ cells (right). c The representative images of blood smear were shown by Wright-Giemsa’s stain when the vehicle mice became moribund (left) and statistical analysis of the percentage of leukemia blasts in the blood (right). Bar represents 10 µm, and these images were amplified 200 fold. d Overall survival was indicated in THP1-GFP-xenografted NSG mice treated with (n = 6) or without WP1130 (n = 6). [file 12967_2020_2384_MOESM7_ESM.tif]
