# Supplementary material for: WT1 facilitates the self-renewal of leukemia-initiating cells through the upregulation of BCL2L2: WT1-BCL2L2 axis as a new acute myeloid leukemia therapy target
Source: J Transl Med. 2020 Jun 24;18:254. doi: 10.1186/s12967-020-02384-y (PMC7313134; doi:10.1186/s12967-020-02384-y)
Supplement: Supplementary file 4 — Additional file 4: Table S2. The sequences of primers for qRT-PCR and construction of plasmids. [file 12967_2020_2384_MOESM4_ESM.docx]

| Genes | Sequences |
| --- | --- |
| WT1-L | 5′-CAA TCA GGG TTA CAG CAC GG-3′ |
| WT1-R | 5′-GCT TGA ATG AGT GGT TGG GG-3′ |
| BCL2L2 | 5′-GCG GAG TTC ACA GCTC TAT AC-3′ |
| BCL2L2 | 5′-AAA AGG CCC CTA CAG TTA CCA-3′ |
| β-actin-L | 5′-TGG CAT CCA CGA AAC TAC CT-3′ |
| β-actin-R | 5′-CGT ACA GGT CTT TGC GGA TG-3′ |
| wt1-L (mouse) | 5′-AAG GAG ACA CAC AGG TGT GAA A-3′ |
| wt1-R (mouse) | 5′-GTG GGT CTT CAG ATG GTC GG-3′ |
| β-actin-L (mouse) | 5′-GGC TGT ATT CCC CTC CAT CG-3′ |
| β-actin-R (mouse) | 5′-CCA GTT GGT AAC AAT GCC ATG T-3′ |
| pCMV-WT1-L* | 5′-CGG AAT TCA CTG CAG GAC CCG GCT TCC-3′ |
| pCMV-WT1-R* | 5′-CCC AAG CTT TCA AAG CGC CAG CTG GAG-3′ |
| pGL3-BCL2L2-L* | 5′-GGG GTA CCC CA AAC CAG AAG TGC TCC TTC-3′ |
| pGL3-BCL2L2-R* | 5′-CCG CTC GAG AGC TGC CTC AGC CTC CAA T-3′ |
| sh-wt1 (mouse)* | 5′-GCA GTG ACA ATT TAT ACC AAA-3′ |

**Table S2: The sequences of primers for qRT-PCR and construction of plasmids**

*Primers for construction of plasmids.
