# Supplementary material for: WT1 facilitates the self-renewal of leukemia-initiating cells through the upregulation of BCL2L2: WT1-BCL2L2 axis as a new acute myeloid leukemia therapy target
Source: J Transl Med. 2020 Jun 24;18:254. doi: 10.1186/s12967-020-02384-y (PMC7313134; doi:10.1186/s12967-020-02384-y)
Supplement: Supplementary file 8 — Additional file 8: Table S3. Limiting dilution assay of MLL-AF9-induced mouse leukemia transduced with sh-nc or sh-wt1. [file 12967_2020_2384_MOESM8_ESM.docx]

**Table S3. Limiting dilution assay of MLL-AF9-induced mouse leukemia transduced with sh-nc or sh-wt1**

| Dose | sh-nc (response/total) | sh-wt1 (response/total) | *P* value |
| --- | --- | --- | --- |
| 30 | 2/6 | 0/6 |  |
| 90 | 5/6 | 3/6 |  |
| 180 | 6/6 | 4/6 |  |
| LSC frequency | 1 in 53 | 1 in 177 | 0.0161 |

The numbers of response mice mean that the recipient mice develop full-blown leukemia and die within 20 weeks after transplantation.
