# Supplementary material for: WT1 facilitates the self-renewal of leukemia-initiating cells through the upregulation of BCL2L2: WT1-BCL2L2 axis as a new acute myeloid leukemia therapy target
Source: J Transl Med. 2020 Jun 24;18:254. doi: 10.1186/s12967-020-02384-y (PMC7313134; doi:10.1186/s12967-020-02384-y)
Supplement: Supplementary file 9 — Additional file 9: Table S4. Limiting dilution assay of MLL-AF9-induced mouse leukemia treated with or without WP1130. [file 12967_2020_2384_MOESM9_ESM.docx]

**Table S4. Limiting dilution assay of MLL-AF9-induced mouse leukemia treated with or without WP1130**

| Dose | Vehicle (response/total) | WP1130 (response/total) | *P* value |
| --- | --- | --- | --- |
| 30 | 3/6 | 0/6 |  |
| 90 | 5/6 | 3/6 |  |
| 180 | 6/6 | 5/6 |  |
| LSC frequency | 1 in 45 | 1 in 139 | 0.0221 |

The numbers of response mice mean that the recipient mice develop full-blown leukemia and die within 20 weeks after transplantation.
